# Heterologous Substitution of *Mycobacterium tuberculosis* rRNA in *Mycobacterium smegmatis* and Its Impact on Antimicrobial Susceptibility

**DOI:** 10.3390/antibiotics15010030

**Published:** 2025-12-31

**Authors:** Qianwen Yue, Chan Shan, Arslan Habib, Guoping Zhao, Xiaoming Ding

**Affiliations:** 1 Collaborative Innovation Center for Genetics and Development, State Key Laboratory of Genetic Engineering, Department of Microbiology, School of Life Sciences, Fudan University, Shanghai 200438, China; 2 Laboratory of Molecular Immunology, State Key Laboratory of Genetic Engineering, School of Life Sciences, Fudan University, Shanghai 200438, China

**Keywords:** antimicrobial susceptibility, *Mycobacterium smegmatis*, *Mycobacterium tuberculosis*, ribosome, rRNA

## Abstract

**Background**: The global incidence of multidrug-resistant and extensively drug-resistant tuberculosis continues to rise. The ribosome serves as a target for multiple antimicrobials, making functional research on it hold great significance. **Methods**: Using homologous recombination combined with a multiple serine integrase-mediated site-specific recombination system, we replaced the two endogenous rRNA operons in *Mycobacterium smegmatis* MC^2^ 155 with a single copy of the single rRNA operon from *Mycobacterium tuberculosis* H37Rv, constructing the *M. smegmatis* BRkoA strain. We assessed growth kinetics at 37 °C, cold sensitivity at lower temperatures, transcriptional levels by RT-qPCR, 70S ribosome integrity through cryo-EM, and antimicrobial susceptibility by microdilution assays. **Results**: The BRkoA strain was successfully constructed. It exhibited markedly slower growth compared to the wild-type strain. Cold-sensitivity assays indicated potential ribosome assembly defects, while transcriptional analysis suggested altered rRNA processing and modification. Cryo-EM analysis further demonstrated the absence of specific ribosomal proteins in the BRkoA 70S ribosome. Moreover, BRkoA displayed reduced susceptibility tendency to several ribosome-targeting antibiotics, including kanamycin, amikacin, paromomycin, gentamicin, and linezolid. **Conclusions**: Replacement of the two endogenous *rrn* operons in *M. smegmatis* with a single copy of the single *M. tuberculosis rrn* operon using a serine integrase-mediated recombination system caused growth impairment and decreased sensitivity tendency to several ribosome-targeting antimicrobials. These findings suggest that ribosome structural variation contributes to intrinsic drug resistance mechanisms.

## 1. Introduction

*Mycobacterium tuberculosis* is the etiological agent of tuberculosis, a disease that remains one of the top 10 causes of infectious mortality worldwide, claiming approximately 1.23 million lives annually [1,2]. Drug-resistant TB has emerged as a major global health challenge with increasing cases of multidrug-resistant (MDR) strains that resist the most effective anti-tuberculosis drugs [2]. Understanding the mechanisms underlying drug resistance and developing new therapeutic strategies are therefore an urgent research priority for TB control.

Ribosomes are the targets of many drugs, such as kanamycin, amikacin, gentamicin, and linezolid, which are amino-glycoside-like antibiotics that acts on ribosomes. Research on the structure and function of ribosomes, especially ribosomes of pathogenic bacteria *M. tuberculosis*, has important implications for developing new anti-tuberculosis drugs and conducting tuberculosis prevention and treatment. At the same time, the ribosome is a highly conserved complex responsible for protein synthesis in all living organisms [3,4,5]. Research on them will also allow us to have a further understanding of the specific details of the pathogen translation process, helping to increase our understanding of the *Mycobacterium* pathogen mechanisms and drug resistance mechanisms [6].

The pathogenic *M. tuberculosis* grows slowly, and its cultivation requires special conditions such as P3 laboratory. The genetic modification methods available for it are also relatively limited [7]. In contrast, *Mycobacterium smegmatis* MC^2^ 155 is a non-pathogenic [8], fast-growing mycobacterial species [9,10] that shares many physiological characteristics with the pathogenic *M. tuberculosis* [11,12]. Consequently, *M. smegmatis* serves as a widely used surrogate model for studying *M. tuberculosis* biology and drug responses [13]. By utilizing synthetic biology approaches, if we take *M. smegmatis* as the host and assemble all the ribosomal genes of *M. tuberculosis* into a functional module to replace the endogenous ribosomes of *M. smegmatis*, it may be possible to construct a new chassis host that better mimics the protein synthesis machinery of *M. tuberculosis*. This would lead to a deeper understanding of ribosomal function in *M. tuberculosis* and hold significant implications for the screening of novel antitubercular drugs and the study of drug resistance mechanisms in *Mycobacterium*.

Bacterial ribosomes consist of ribosomal proteins (RPs) and ribosomal RNA (rRNA), forming the 70S particle composed of a 50S large subunit and a 30S small subunit. The 30S subunit contains the 16S rRNA and over 21 RPs, while the 50S subunit contains 5S rRNA and 23S rRNA along with more than 34 RPs [14,15,16]. RPs assist in rRNA folding and structural stability, whereas rRNA plays central roles in mRNA and tRNA recognition and catalysis during protein synthesis. The number of rRNA (*rrn*) operons is species-specific: *M. smegmatis* contains two *rrn* operons (*rrnA* and *rrnB*), whereas *M. tuberculosis* has only one. Comparative sequence analysis showed that one *M. smegmatis* operon closely resembles the *rrnA*_s_ of slow-growing mycobacteria and was designated *rrnA*_f_ (“f” for fast-grower), while the second operon, *rrnB*_f_, is more distantly related to the slow-grower *rrnA*_s_ operon [17].

The aim of this study is to investigate whether a single copy of the single *rrn* operon from *M. tuberculosis* can fully substitute the two endogenous *rrn* operons from *M. smegmatis* strains functionally, and its impact on antimicrobial susceptibility.

## 2. Results

### 2.1. Construction and Validation of M. smegmatis BRkoA Strain

**Figure 1:**
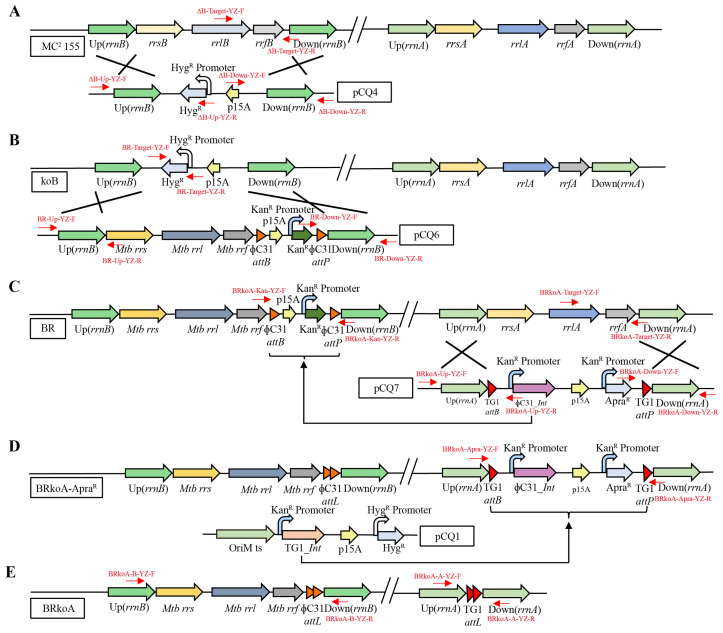
Construction workflow of the *M. smegmatis* BRkoA strain. (**A**) The *rrnB* of *M. smegmatis* MC^2^ 155 was disrupted via homologous recombination, generating the koB strain carrying hygromycin B resistance. (**B**) The disrupted operon was complemented with the *rrn* operon from *M. tuberculosis*, producing the BR strain containing one native *rrnA* operon and one *M. tuberculosis rrn* operon, along with kanamycin resistance. (**C**) The remaining native *rrnA* operon in the BR strain was deleted, and kanamycin resistance was excised, resulting in the BRkoA-Apra^R^ strain carrying apramycin resistance. (**D**) The apramycin resistance marker was removed and an exit plasmid was introduced, generating the BRkoA-Hyg^R^ strain with hygromycin B resistance. (**E**) The exit plasmid was cured by elevating the culture temperature, yielding the final BRkoA strain with no antibiotic resistance markers. Red arrows indicate primer positions used for strain validation.

To construct the *M. smegmatis* BRkoA strain, we employed an efficient circular gene knockout system [18] combined with homologous recombination and a multiple serine integrase-mediated site-specific recombination strategy [19] to replace both endogenous *rrn* operons of *M. smegmatis* MC^2^ 155 with the *rrn* operon from *M. tuberculosis* H37Rv. A schematic overview of the strain construction workflow is shown in Figure 1. In the first step, *M. smegmatis* MC^2^ 155 was transformed with plasmid pCQ4 to disrupt the native *rrnB* operon, generating the knockout strain koB (Figure 1A). In the second step, plasmid pCQ6 was introduced into the koB strain, resulting in strain BR, which contained one native *rrnA* operon and one *M. tuberculosis rrn* operon (Figure 1B). Subsequently, plasmid pCQ7 was used to replace the remaining native *rrnA* operon, yielding the BRkoA-Apra^R^ strain that carried only the *M. tuberculosis rrn* operon (Figure 1C). In the fourth step, pCQ1 was introduced to excise the antibiotic resistance cassette, producing the BRkoA-Hyg^R^ strain (Figure 1D). Finally, heat-induced plasmid curing was performed to remove pCQ1, generating the final BRkoA strain, which harbors the *M. tuberculosis rrn* operon without any antibiotic marker (Figure 1E). Together, these genetic manipulations successfully replaced both endogenous *rrn* operons of *M. smegmatis* with the *M. tuberculosis rrn* operon, resulting in the construction of the marker-free BRkoA strain.

**Figure 2:**
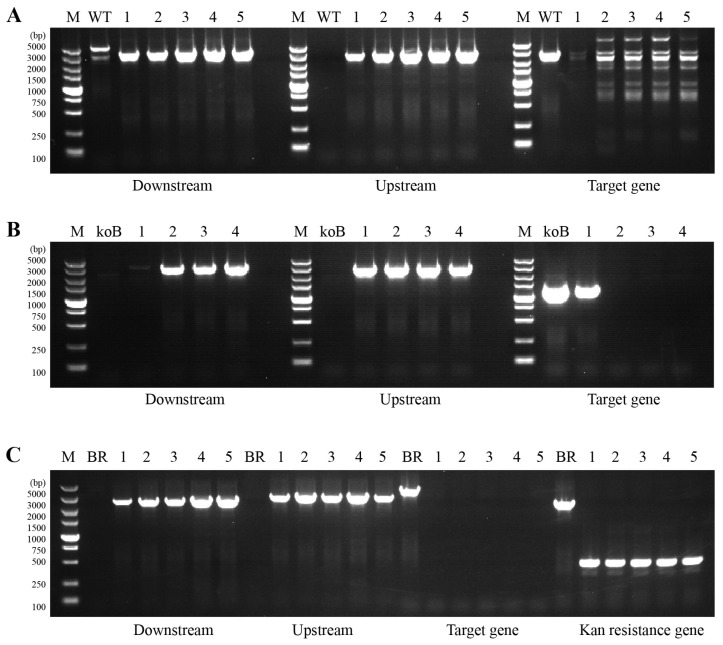
Validation of *M. smegmatis* MC^2^ 155 derivative strains by PCR analysis. (**A**) PCR verification of *rrnB* knockout (koB) strains. Lanes 1–5 correspond to individual knockout clones. The expected PCR fragment sizes for the downstream homologous arm, upstream homologous arm, and target gene regions are 3144 bp, 2785 bp, and 2499 bp, respectively. (**B**) PCR verification of *rrn* operon complementation in BR strains. Lanes 1–4 represent independent complemented clones. The expected PCR fragment sizes for the downstream homologous arm, upstream homologous arm, and target gene regions are 3065 bp, 2819 bp, and 1186 bp, respectively. (**C**) PCR verification of *rrnA* knockout in the complemented BR background (BRkoA-Apra^R^ strains). Lanes 1–5 correspond to verified knockout clones. The expected PCR fragment sizes for the downstream homologous arm, upstream homologous arm, target gene, and kanamycin resistance gene (present or excised) are 2523 bp, 2862 bp, 3826 bp, and 2409 bp or 456 bp, respectively. All positive PCR products were confirmed by Sanger sequencing and compared using SnapGene v3.2.1.

The *M. smegmatis* derivative strains were verified through PCR amplification using primer sets targeting the upstream and downstream homologous arm exchange regions, internal regions of the target genes, and both ends of the integrase recognition sites. Compared with the wild-type (WT) strain, PCR analysis confirmed successful deletion of the *rrnB* operon in *M. smegmatis* MC^2^ 155 for knockout clones 1 to 5 (Figure 2A). When compared with the *rrnB* knockout (koB) strain, the *rrn* operon from *M. tuberculosis* H37Rv was successfully integrated and complemented in BR strains 2 to 4, as verified by PCR amplification (Figure 2B). Subsequently, deletion of the *rrnA* operon from *M. smegmatis* MC^2^ 155 in the complemented BR background was achieved, resulting in the BRkoA-Apra^R^ strains (clones 1–5), which were validated by PCR using the same primer strategy (Figure 2C).

**Figure 3:**
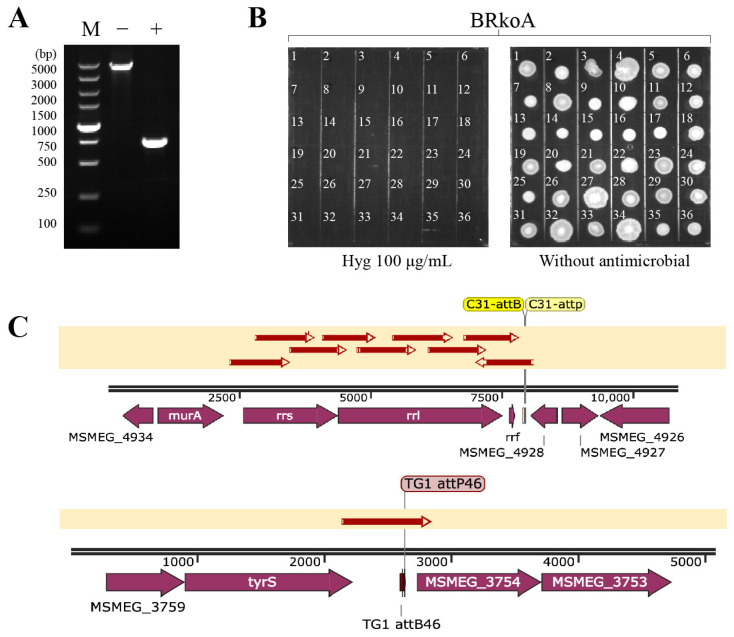
Verification of the BRkoA strain construction and marker excision. (**A**) PCR verification of the BRkoA-Hyg^R^ strain following apramycin resistance marker removal. The expected PCR fragment sizes for the Apra^R^ gene (present) and after excision (absent) are 4605 bp and 745 bp, respectively. (**B**) Confirmation of plasmid curing in the BRkoA strain. BRkoA clones were tested for growth on 7H10 agar with or without hygromycin B to verify the loss of the exit plasmid. (**C**) Sanger sequencing confirmation of the BRkoA strain. Lane M: DL 5000 DNA ladder; Lane WT: PCR product of wild-type *M. smegmatis* MC^2^ 155. All positive PCR products were confirmed by Sanger sequencing and compared using SnapGene v3.2.1.

Following the removal of the apramycin resistance cassette, PCR analysis confirmed successful excision of the Apra^R^ marker in the BRkoA-Hyg^R^ strain (Figure 3A). The elimination of hygromycin resistance was further verified phenotypically: BRkoA colonies (1–36) were able to grow on 7H10 agar plates without antibiotics but failed to grow on plates supplemented with hygromycin B (Figure 3B), confirming the loss of hygromycin resistance. Finally, the correct genomic arrangement of the BRkoA strain was validated by Sanger sequencing, confirming precise recombination at the expected loci (Figure 3C). Together, these results demonstrate the successful construction of an *M. smegmatis* BRkoA strain harboring a heterologous *M. tuberculosis rrn* operon, thereby generating a strain with hybrid ribosomal composition.

### 2.2. Growth Characteristics of the M. smegmatis BRkoA Strain

**Figure 4:**
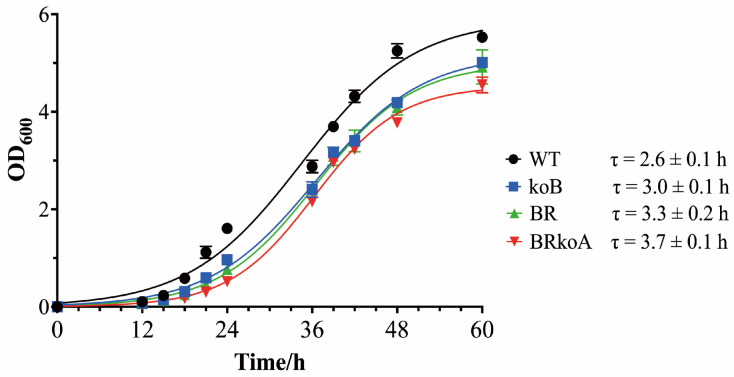
Growth dynamics of the wild-type *M. smegmatis* MC^2^ 155 and *rrn*-modified derivative strains. OD_600_ values were recorded over time in 7H9 medium. Data represent the means from three biological replicates, with error bars showing standard deviations. τ indicate doubling time of the strains.

To evaluate the impact of *rrn* replacement on cellular growth, we monitored the OD_600_ of all strains cultured in 7H9 medium. The WT strain exhibited the fastest growth, reaching stationary phase earlier and achieving the highest final biomass. Both koB and BR strains demonstrated moderate growth attenuation relative to WT but were nearly indistinguishable from each other, indicating that the presence of a single *rrnA* operon either alone (koB) or coexisting with an additional *M. tuberculosis rrn* operon (BR) is sufficient to maintain near-normal growth (Figure 4).

In contrast, the BRkoA strain grew substantially more slowly across all time points. Because BRkoA depends exclusively on the *M. tuberculosis rrn* operon for rRNA synthesis, these data suggest that the heterologous *rrn* operon alone cannot fully support optimal ribosome production and cellular proliferation in *M. smegmatis*.

### 2.3. Transcriptional Level of M. smegmatis BRkoA Strain

**Figure 5:**
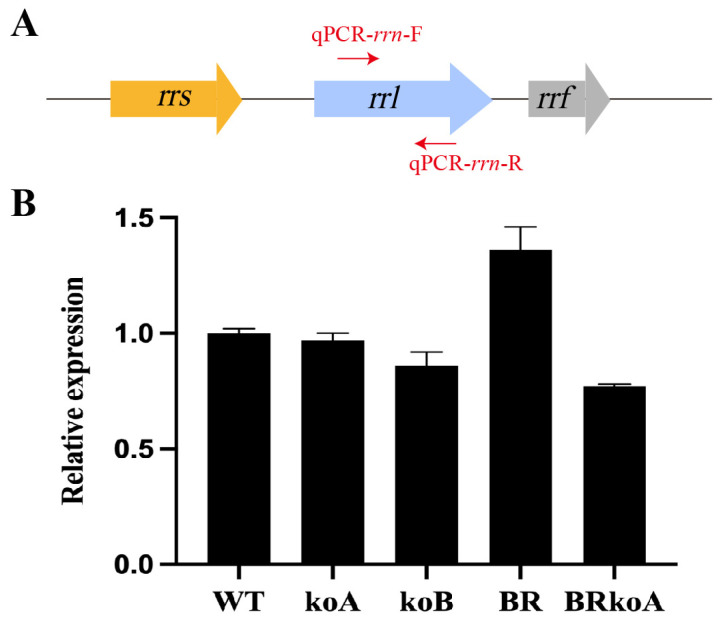
Transcriptional analysis of the *M. smegmatis* MC^2^ 155 strain and its derived *rrn*-modified strains. (**A**) Schematic representation of the primer design used to distinguish transcripts originating from the *M. smegmatis* and *M. tuberculosis rrn* operons. (**B**) The relative transcription levels of *rrn* measured using primers targeting a conserved region of *rrl*. All experiments were performed in three independent biological replicates, and error bars represent the standard deviations of the means.

The *rrn* between *M. smegmatis* MC^2^ 155 and *M. tuberculosis* H37Rv share high sequence identity (~91.2%), primers (qPCR-*rrn*-F and qPCR-*rrn*-R) targeting a conserved region of *rrl* shared by both species were designed to assess the rRNA level (Figure 5A). qPCR analysis revealed distinct expression patterns in WT and *rrn*-modified derivative strains (Figure 5B). After normalizing to WT, rRNA levels were slightly reduced in koB (0.86-fold) and marginally reduced in koA (0.97-fold), which only contained one native operon. In contrast, rRNA levels were modestly elevated in BR (1.36-fold), which contained the native *M. smegmatis rrnA* operon and the *M. tuberculosis rrn* operon.

Notably, BRkoA exhibited a moderate reduction in rRNA level (0.77-fold) compared to WT and koA, consistent with the slowed growth phenotype and suggesting that the heterologous *rrn* operon alone does not fully sustain the optimal rRNA supply required for rapid proliferation.

### 2.4. Cold Sensitivity of M. smegmatis BRkoA Strain

**Figure 6:**
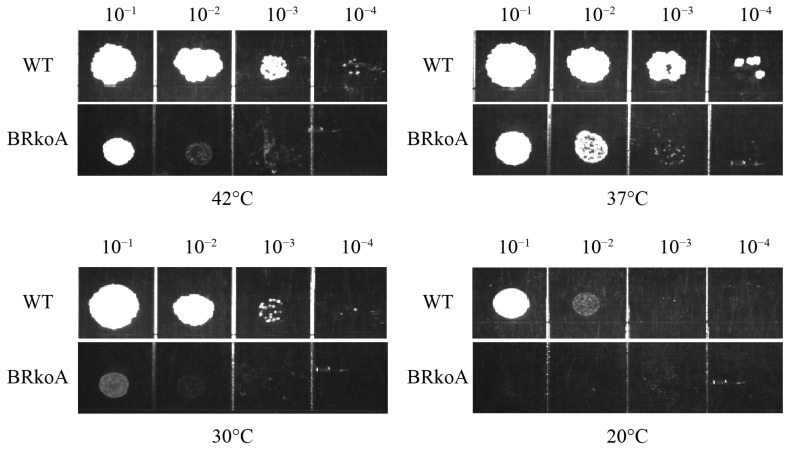
Cold sensitivity of cells expressing wild-type and BRkoA ribosomes. Serial dilution spot assays of WT and BRkoA *M. smegmatis* strains grown at 42 °C, 37 °C, 30 °C, and 20 °C. The BRkoA strain shows reduced growth at non-optimal temperatures, indicating a temperature-sensitive phenotype consistent with impaired ribosome function.

Mutations that impair ribosome assembly often confer temperature-sensitive growth defects, particularly at lower temperatures [20,21]. To determine whether the BRkoA affects ribosome biogenesis, we evaluated the temperature-dependent growth of the BRkoA and WT *M. smegmatis* using serial dilution spot assays. Cultures were incubated at 42 °C, 37 °C, 30 °C, and 20 °C (Figure 6).

At the best growth temperature of 37 °C, BRkoA already exhibited a slight reduction in colony formation compared with WT. Growth impairment became more pronounced at non-optimal temperatures. At 30 °C, BRkoA displayed a substantial decrease in viability, whereas at 20 °C, growth was nearly abolished, indicating a strong cold-sensitive phenotype. A large reduction in growth was also observed at 42 °C, suggesting sensitivity to both heat and cold stress. These findings indicate that rRNA is important for maintaining normal ribosome function across temperature ranges, and the pronounced cold sensitivity of the mutant is consistent with a defect in ribosomal assembly or maturation.

### 2.5. 70S Ribosome Structure of the M. smegmatis BRkoA Strain

To assess whether heterologous replacement of the *rrn* operon alters ribosome architecture in Mycobacterium, we visualized the 70S ribosomes from the *M. smegmatis* BRkoA strain. Sucrose gradient analysis of BRkoA showed that at a high Mg^2+^ concentration most of the ribosomal material sedimented as a 70S peak and a 50S peak (Figure S1). Ribosomes of the 70S peak were collected from sucrose gradients and analyzed by cryogenic electron microscopy (cryo-EM). The structure of the BRkoA 70S ribosome was resolved at 2.8 Å, allowing comparison with previously determined ribosome structures.

**Figure 7:**
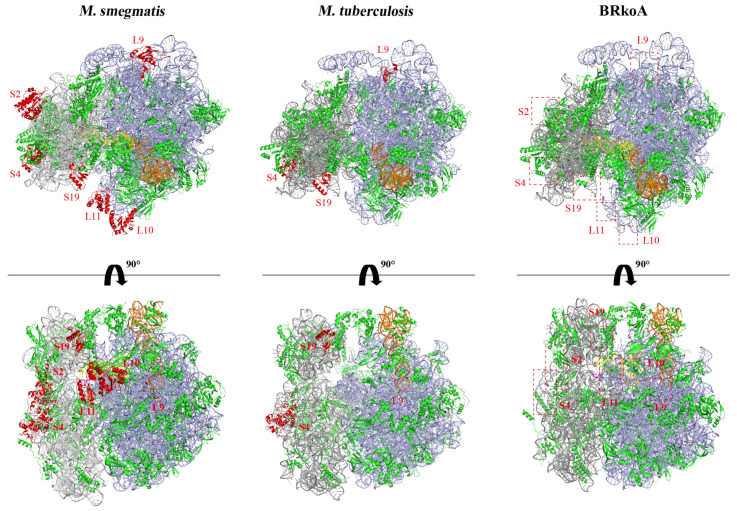
The structure of the *Mycobacterium* 70S ribosome. (**Left**) The *M. smegmatis* ribosome structure [14] (Protein Data Bank (PDB) ID: 5O61). (**Middle**) The *M. tuberculosis* ribosome [15] (PDB ID: 5V93). (**Right**) The BRkoA ribosome. Ribosomal proteins L9, L10, L11, S2, S4, and S19 are highlighted in bright red, and positions where these proteins are missing in BRkoA are indicated by red dashed boxes. The remaining ribosomal proteins in green, 23S rRNA is shown in blue-purple, 16S rRNA in gray, 5S rRNA in orange, tRNA in yellow, mRNA in rose red.

Relative to the native *M. smegmatis* 70S ribosome, several ribosomal proteins were absent in the BRkoA map, including L9, L10, and L11 on the large subunit and S2, S4, and S19 on the small subunit. When compared with the *M. tuberculosis* 70S ribosome, the BRkoA structure similarly lacked L9, S4, and S19. These missing densities correspond to flexible peripheral proteins and may reflect either increased disorder or reduced occupancy in the engineered ribosomes (Figure 7). Overall, the structural analysis indicates that the heterologous *M. tuberculosis*-derived ribosome expressed in the BRkoA strain is highly similar to the native *M. tuberculosis* 70S ribosome, while differing more substantially from the endogenous *M. smegmatis* ribosome.

### 2.6. Susceptibility Profiles of M. smegmatis BRkoA Strain

To assess whether heterogeneous rRNA influence the susceptibility of the *M. smegmatis* BRkoA strain to ribosome-targeting antimicrobial agents, we first determined the minimal inhibitory concentrations (MICs) for *M. smegmatis* MC^2^ 155 and BRkoA. The MICs for MC^2^ 155 were: 1 μg/mL for kanamycin, 1 μg/mL for amikacin, 2 μg/mL for paromomycin, 2 μg/mL for gentamicin, and 2 μg/mL for linezolid. In comparison, the BRkoA strain exhibited MICs of 2 μg/mL for kanamycin, 1 μg/mL for amikacin, 4 μg/mL for paromomycin, 4 μg/mL for gentamicin, and 4 μg/mL for linezolid (Table 1).

**Figure 8:**
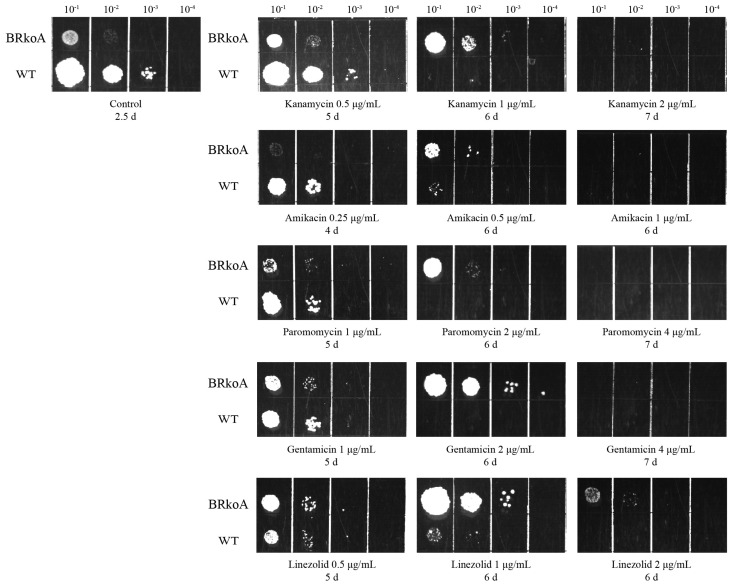
The *M. smegmatis* BRkoA strain exhibits reduced sensitivity tendency to kanamycin, amikacin, paromomycin, gentamicin, and linezolid.

We next compared the growth of *M. smegmatis* MC^2^ 155 and BRkoA across serial dilutions when exposed to each antibiotic (Figure 8). Under control conditions, both strains grew comparably at all dilutions. However, upon antibiotic exposure, BRkoA consistently showed greater survival than WT, particularly at higher concentrations and longer incubation times. For example, WT growth was nearly abolished at 1 μg/mL kanamycin by day 6, whereas BRkoA retained visible colonies at 10^−2^ and 10^−3^ dilutions. A similar trend was observed for amikacin, paromomycin, gentamicin, and linezolid: at concentrations corresponding to or exceeding the WT MICs, BRkoA maintained growth at multiple dilutions where WT growth was completely inhibited. These results demonstrated that the BRkoA strain exhibits reduced sensitivity tendency to kanamycin, amikacin, paromomycin, gentamicin, and linezolid, consistent with the elevated MICs observed.

## 3. Discussion

The ribosome is composed of ribosomal proteins and rRNA, and rRNA serving as the structural scaffold of the ribosome plays an essential role in its architecture and function [22]. In this study, we used a homologous recombination strategy combined with a multiple serine integrase-mediated site-specific recombination system to construct the *M. smegmatis* BRkoA strain, which contains a heterologous ribosome composed of *M. tuberculosis* H37Rv rRNA and *M. smegmatis* MC^2^ 155 ribosomal proteins [18]. By comparing the growth curves of the koB, BR, and BRkoA strains, we found that both deletion of the native *rrn* operon in *M. smegmatis* and complementation with the *M. tuberculosis rrn* operon significantly affected bacterial growth.

The koB strain exhibited slower growth, possibly due to differences in promoter number and regulatory strength between the *rrnB* and *rrnA* operons. The *rrnB* operon is driven by three promoters, whereas *rrnA* has only one [17]. With only the *rrnA* operon remaining in the koB strain, transcriptional regulation becomes limited, insufficient to maintain rRNA levels comparable to the WT strain, resulting in reduced growth. Complementation with the *M. tuberculosis rrn* operon in the BR strain did not restore growth to wild-type levels; instead, its growth resembled that of koB. This may reflect suboptimal assembly or functionality of the heterologous ribosomes, leading to reduced fitness relative to WT. When the *M. smegmatis rrnA* operon was additionally deleted to generate the BRkoA strain, growth was further impaired, indicating that dependence on the heterologous *M. tuberculosis* rRNA alone exacerbates the growth defect.

The operons encode large primary transcripts that are processed and chemically modified, and then assembled into ribosomes with ribosomal proteins in a multi-step process [23]. To further investigate the relative expression of the *rrn* operons, we used qPCR primers designed within a homologous region of *rrl* shared by both species, the rRNA levels were slightly reduced in koB (0.86-fold) and modestly elevated in BR (1.36-fold). Notably, BRkoA exhibited a moderate reduction in rRNA level (0.77-fold) compared to WT and koA, indicating that heterologous substitution of rRNA altered rRNA processing.

Defects in ribosome biogenesis are frequently associated with cold-sensitive phenotypes [20,21]. Consistent with this, the *M. smegmatis* BRkoA strain harboring heterogeneous rRNA exhibited growth at 37 °C but failed to form colonies at 30 °C. This cold sensitivity suggests that ribosomes assembled from *M. tuberculosis* rRNA and *M. smegmatis* ribosomal proteins may be compromised in assembly or stability under sub-optimal temperatures. To further investigate these defects, we examined the structure of the 70S ribosome from the BRkoA strain using cryo-EM. Compared with the *M. smegmatis* 70S structure, ribosomal proteins L9, L10, L11, S2, S4, and S19 were absent in the BRkoA ribosome. When compared with the *M. tuberculosis* 70S ribosome, proteins L9, S4, and S19 were absent. These structural differences indicate incomplete or altered assembly of the heterologous ribosome. Nevertheless, the overall architecture of the BRkoA 70S ribosome more closely resembled that of *M. tuberculosis*, consistent with its rRNA origin. Mass-spectrometry can be used for further quantitative proteomics analysis the presence of individual ribosomal proteins in BRkoA.

The ribosome is one of the primary targets of antibacterial agents, with many antibiotics exerting their effects by interfering with either the small 30S or the large 50S subunit [24,25]. In this study, we observed that the *M. smegmatis* BRkoA strain exhibited reduced sensitivity tendency to kanamycin, amikacin, paromomycin, gentamicin, and linezolid. Kanamycin, amikacin, paromomycin, and gentamicin belong to the aminoglycoside class. Kanamycin exerts an influence that diminishes the accuracy of mRNA translation by interacting with the 16S rRNA helix 44 [26]. Amikacin is derived of kanamycin, interacts with the 30S small subunit and suppresses the assembly of the initiation complex; additionally, it promotes mistranslation during the elongation phase and blocks peptide release during termination, thereby preventing ribosome recycling [27]. Paromomycin and gentamicin also bind near the top of helix 44, inducing conformational rearrangements of A1492 and A1493 that facilitate binding of non-cognate tRNAs to the A site and increase miscoding [28]. Linezolid, an oxazolidinone antibiotic, binds to a pocket within domain V of the 23S rRNA peptidyl transferase center and prevents peptide transfer during protein synthesis [14]. Given these mechanisms, the reduced susceptibility tendency of the BRkoA strain suggests that the heterologous ribosome composed of *M. tuberculosis* rRNA assembled with *M. smegmatis* ribosomal proteins undergoes structural alterations that may impact antibiotic binding sites. Such changes in spatial conformation could diminish the ability of multiple ribosome-targeting antibiotics to correctly recognize or interact with their binding regions, thereby contributing to the observed phenotype of reduced drug sensitivity tendency. Amikacin is derived of kanamycin, obtained through acetylation with the L(-)-gamma-amino-alpha-hydroxybutyryl side chain at the C-1 amino group of the deoxystreptamine moiety and its antibacterial activity is generally equal to or greater than that of kanamycin against sensitive organisms [29]. The MIC of amikacin in BRkoA remained unchanged, likely due to its alpha-hydroxyl group and terminal basic function in the side chain overcoming modifications and maintain stable target binding.

The possession of a fully assembled ribosome containing all ribosomal proteins and rRNAs are critical for bacterial survival and function [23,30]. As the core machinery for protein synthesis, the ribosome’s integrity directly determines a bacterium’s ability to efficiently and accurately translate mRNA into functional protein. A complete ribosome comprises not only rRNA and ribosomal proteins but also requires precise processing and assembly steps to form an active structure. Defects in rRNA process can disrupt ribosome assembly, leading to reduced protein synthesis rates, slowed cell growth, and even metabolic dysregulation or impaired stress responses. The BRkoA strain grew substantially more slowly compared to WT, therefore necessitates sufficient pre-experimental incubation time to achieve appropriate cell density. Notably, the parental strain of BRkoA is non-pathogenic, and the engineering only heterologous substitution of rRNA without introducing virulence genes, ensuring strain safety. Furthermore, antibiotic susceptibility experiments further demonstrate that BRkoA serves as a promising novel research chassis, exhibiting significant potential in drug screening. Looking forward, the BRkoA strain is expected to become a tool used for the high-throughput discovery of ribosome-targeting antibiotics. Expanding the scope, the heterologous substitution of rRNA along with the strategies presented herein might act as alternative entry points for uncovering and facilitating the laboratory evolution of unprecedented translational properties.

## 4. Materials and Methods

### 4.1. Bacterial Strains and Culturing Conditions

The characteristics of the bacterial strains used in this study are listed in Table S1. *Escherichia coli* DH10B was grown either in Luria–Bertani (LB) broth or on LB solid media at 37 °C for 12–16 h. *M. smegmatis* MC^2^ 155 and its derivative strains were grown either in Middlebrook 7H9 broth (BD Difco, Sparks, MD, USA) supplemented with 10% OADC (Oleic Albumin Dextrose Catalase) growth supplement (100 mL OADC containing 12 µL oleic acid, 5 g albumin, 2 g glucose, 0.03 g catalase, and 0.85 g NaCl) and 0.05% Tween-80 at 37 °C for 1–3 days or on Middlebrook 7H10 solid media (BD Difco, Sparks, MD, USA) supplemented with 10% OADC and 0.4% glycerol at 37 °C for 2–7 days. The gene *SacB* was employed for negative selection, and 10% sucrose was added to 7H9-OADC broth or 7H10-OADC solid media when required. Antibiotic was added to LB broth, LB solid media, 7H9-OADC broth or 7H10-OADC solid media at the following final concentrations when appropriate: kanamycin (50 µg/mL), ampicillin (100 µg/mL), hygromycin B (100 µg/mL), and apramycin (50 µg/mL), respectively.

### 4.2. Construction of Plasmids

The plasmids used in this study are summarized in Table S2, and the primers are listed in Table S3. The construction of plasmids was carried out as follows. First, target PCR products list in Table S3 were amplified using specific primers and High-Fidelity DNA Polymerase (Vazyme, Nanjing, China) following the manufacturer’s instructions on Veriti^TM^ (Applied Biosystems, Waltham, MA, USA). Products pCQ1-oriMts and pCQ1-TGint-ori p15A-Hyg were used to construct pCQ1; pCQ4-ΔB-left flank, pCQ4-ΔB-right flank, pCQ4-ΔB-*SacB*, pCQ4-ΔB-Amp, pCQ4-ΔB-*ori* p15A&Hyg were used to construct pCQ4; pCQ6-BR-left flank, pCQ6-BR-right flank, pCQ6-BR-*SacB*, pCQ6-BR-Mt *rrn*, pCQ6-BR-*ori* p15A&Kan were used to construct pCQ6; pCQ7-ΔA-left flank, pCQ7-ΔA-right flank, pCQ7-ΔA-*SacB*&Amp, pCQ7-ΔA-ϕC31-*ori* p15A&Apar were used to construct pCQ7. PCR products were then analyzed by agarose gel electrophoresis and purified using an Agarose Gel DNA Extraction Kit (Vazyme, Nanjing, China) following the manufacturer’s instructions. Next, purified PCR fragments were seamlessly ligated using a NovoRec^®^ plus One step PCR Cloning Kit (Novoprotein, Suzhou, China) and subsequently transformed into *E. coli* DH10B competent cells following the manufacturer’s instructions. The plasmids were extracted from bacterial clones using a Plasmid Preparation Kit and the recombinant constructs were verified by restriction endonuclease digestion (Thermo Fisher Scientific, Waltham, MA, USA) and confirmed through Sanger sequencing (Tsingke, Beijing, China).

### 4.3. Bacterial Growth Curve

*M. smegmatis* BRkoA and MC^2^ 155 control strains were initially cultured on 7H10-OADC plates and incubated at 37 °C until visible colonies appeared (approximately 3 days). Then, individual colonies were selected and inoculated into 3 mL of 7H9-OADC liquid medium, which was then cultured for 1–2 days to generate seed cultures. Each seed culture was subsequently diluted with 7H9-OADC medium to an OD_600_ of 1. The diluted cultures were further inoculated at 1% (*v*/*v*) into 100 mL of 7H9-OADC medium in conical flasks, with each experimental group performed in biological triplicate. The OD_600_ of each culture was measured 12 h post-inoculation, then every 3 h during logarithmic growth, every 6 h in the late logarithmic phase, and every 12 h upon reaching the stationary phase. Growth data were fitted using the logistic growth model in GraphPad PRISM 8.0.2. Growth curves were plotted with time as the x-axis and the mean OD_600_ of each group as the y-axis. The doubling time of each culture was calculated using the Growthcurver package v0.3.0 in R v3.5.2.

### 4.4. Cold Sensitivity Experiment

The growth patterns of the *M. smegmatis* MC^2^ 155 and BRkoA were compared by monitoring OD_600_ at regular intervals. For the cold sensitivity assay, mid-log phase cultures (OD_600_ ≈ 1.0) were harvested and diluted 1:10 with 1× PBS to achieve an OD_600_ of 0.1. Subsequently, 100 μL dilution was further serially diluted by 900 μL PBS to prepare 10-fold serial dilutions ranging from 10^−1^ to 10^−4^. Aliquots of 5 μL from each dilution were then spotted on 7H10 plates and incubated at 42 °C, 37 °C, 30 °C, and 20 °C for 3 days.

### 4.5. RT-qPCR

Primers used for qPCR are listed in Table S4. To assess the transcript levels of *rrn*, *M. smegmatis* MC^2^ 155 and BRkoA were grown at 37 °C in Middlebrook 7H9 medium until reaching an OD_600_ of 0.8. Total RNA was extracted using RNAprep Pure Cell/Bacteria Kit (TIANGEN, Beijing, China). Briefly, 20 mL of bacterial culture was harvested by centrifugation at 4500 rpm for 10 min, and the pellet was resuspended in 1 mL of lysis buffer RL on ice. Cells were disrupted using acid-washed glass beads (Sigma-Aldrich, St. Louis, MO, USA) in a Tissue Lyser (Wonbio, Shanghai, China) at 50 Hz for 60 s, followed by cooling at −80 °C for 60 s. This cycle was repeated six times to ensure thorough cell lysis. Subsequent steps were performed to obtain RNA following the manufacturer’s protocol, taking care to avoid RNase contamination. RNA amount and purity was checked using Nano drop 2000 (Thermo Fisher Scientific, Waltham, MA, USA).

cDNA synthesis was performed using HiScript Q RT SuperMix for PCR (Vazyme, Nanjing, China) following the manufacturer’s instructions. RT-qPCR was carried out using HiScript III RT SuperMix for qPCR (+gDNA wiper) (Vazyme, Nanjing, China) on a Bio-Rad Real-Time PCR System (Bio-Rad, Hercules, CA, USA). The qPCR program was 95 °C for 30 s, followed by 40 cycles (95 °C for 10 s, 60 °C for 30 s), then 95 °C for 15 s, 60 °C for 5 s, 95 °C for 15 s. Each reaction was performed in duplicate using three independent cDNA preparations. Gene expression levels were normalized to the housekeeping gene *sigA*, and relative expression was calculated using the 2^−ΔΔCt^ method.

### 4.6. Sucrose Gradient Analysis of Ribosomes

Bacterial cells were harvested from 200 mL cultures at OD_600_ ≈ 1.0 by centrifugation at 4000× *g* for 5 min at 4 °C. Cell pellets were resuspended in 30 mL of ice-cold resuspension buffer (20 mM Tris-HCl, pH 8.0, 100 mM NH_4_Cl, 12 mM MgCl_2_, and 6 mM β-mercaptoethanol) and collected again by centrifugation at 4000× *g* for 15 min at 4 °C. Pellets were then resuspended in 1 mL of ice-cold lysis buffer (1 mM PMSF, 0.5 U/mL RNase inhibitor and 10 U/mL DNase I).

Cells were disrupted using acid-washed glass beads (Sigma-Aldrich, St. Louis, MO, USA) in a Tissue Lyser (Wonbio, Shanghai, China) at 70 Hz for 60 s, followed by cooling at −80 °C for 60 s. This cycle was repeated six times to ensure thorough lysis. Lysates were clarified by centrifugation at 15,000× *g* for 15 min at 4 °C. The supernatants were loaded onto 12 mL of 10–50% linear sucrose gradients prepared in resuspension buffer. Gradients were centrifuged at 39,000 rpm for 4 h at 4 °C in an SW41 rotor (Beckman Coulter, Brea, CA, USA), and fractionated using a gradient fractionator (BioComp, Fredericton, NB, Canada). Fractions containing 70S ribosomes were pooled, buffer-exchanged into ice-cold resuspension buffer, and stored at −80 °C.

### 4.7. Cryo-EM Analysis of 70S Ribosomes

Cryo-EM grids were prepared by applying 5 μL of sample onto freshly glow-discharged R2/1 holey carbon grids (Quantifoil Micro Tools, Jena, Germany) and allowing the sample to adsorb for 8 s in a chamber maintained at 100% humidity and 4 °C. Grids were then blotted and plunge-frozen into liquid ethane cooled by liquid nitrogen using a Vitrobot Mark IV (Thermo Fisher Scientific, Waltham, MA, USA).

Frozen grids were loaded into a Titan Krios G3i transmission electron microscope (Thermo Fisher Scientific, Waltham, MA, USA). Data acquisition was performed using EPU software (v2.2.2.10REL), collecting image stacks of 40 frames at a total electron dose of 50 e^−^/Å^2^, a defocus range of −0.5 to −2.5 μm, and a total exposure time of 3.02 s.

Raw micrographs were motion-corrected using MotionCor2 to compensate for drift and beam-induced motion [31]. Contrast transfer function (CTF) parameters were estimated using Gctf_v1.06. Subsequent data processing, including several iterations of 2D and 3D classification, was performed in cryoSPARC v2, followed by masked local refinement in RELION v3.0 to generate the final 3D reconstruction [32,33].

For model building, the structure of *M. smegmatis* and *M. tuberculosis* ribosome were rigid-body docked into the cryo-EM density using UCSF Chimera v1.14 [34]. The model was further adjusted in COOT and real-space refined in Phenix v1.19.2 [35]. Figures were generated using PyMOL v2.4.1.

### 4.8. Antimicrobial Susceptibility Testing

The seed culture underwent dilution to achieve an OD_600_ of approximately 0.1. MICs were determined using the microdilution method [36]. Briefly, a volume of 50 μL of bacterial suspension, with an approximate concentration of 5 × 10^5^ CFU, was put into each well of sterile, flat-bottom 96-well plates (Sangon Biotech, Shanghai, China) containing antimicrobial agents at 2-fold serial dilutions. The agents tested included kanamycin, amikacin, paromomycin, gentamicin, and linezolid, with concentrations ranging from 0.125 to 64 μg/mL. Plates were incubated at 37 °C for 1.5 days, with growth in wells without antimicrobial agents serving as the positive control.

For the dilution dot assay, seed cultures (OD_600_~0.1) were further diluted in 10^−1^ to 10^−4^ gradients. Aliquots of 5 μL from each dilution were spotted onto plates with or without antimicrobial agents at various concentrations. Plates were incubated until colonies emerged.

## 5. Conclusions

We successfully constructed the *M. smegmatis* BRkoA strain harboring a heterologous ribosome, in which the two endogenous *rrn* operons of *M. smegmatis* MC^2^ 155 were replaced with a single copy *rrn* operon from *M. tuberculosis* H37Rv. The BRkoA strain exhibited ribosome assembly defects, likely due to altered rRNA processing and the absence of specific ribosomal proteins. Moreover, BRkoA displayed reduced sensitivity tendency to multiple antibiotics, including kanamycin, amikacin, paromomycin, gentamicin, and linezolid.

## Figures and Tables

**Table 1:** Determination of MICs of WT and BRkoA strain.

Antimicrobial Agents	WT MIC (μg/mL)	BRkoA MIC (μg/mL)
Kanamycin	1	2
Amikacin	1	1
Paromomycin	2	4
Gentamicin	2	4
Linezolid	2	4

## Data Availability

The original contributions presented in this study are included in the article and Supplementary Materials. Further inquiries can be directed to the corresponding author.

## Supplementary Materials

The following supporting information can be downloaded at: https://www.mdpi.com/article/10.3390/antibiotics15010030/s1, Figure S1: Sucrose gradient separation of the ribosomal material from *M. smegmatis* BRkoA, Table S1: Strains used in this study; Table S2: Plasmids used in this study; Table S3: Primers used in this study for construct the corresponding plasmids or Sanger sequencing; Table S4: Primers used in this study for construct the corresponding strains or Sanger sequencing.
