# Supplementary material for: Heterologous Substitution of Mycobacterium tuberculosis rRNA in Mycobacterium smegmatis and Its Impact on Antimicrobial Susceptibility
Source: Antibiotics (Basel). 2025 Dec 31;15(1):30. doi: 10.3390/antibiotics15010030 (PMC12838053; doi:10.3390/antibiotics15010030)
Supplement: Supplementary file 1 [file antibiotics-15-00030-s001.zip › antibiotics-4053115-supplementary.pdf]

Supplementary Information

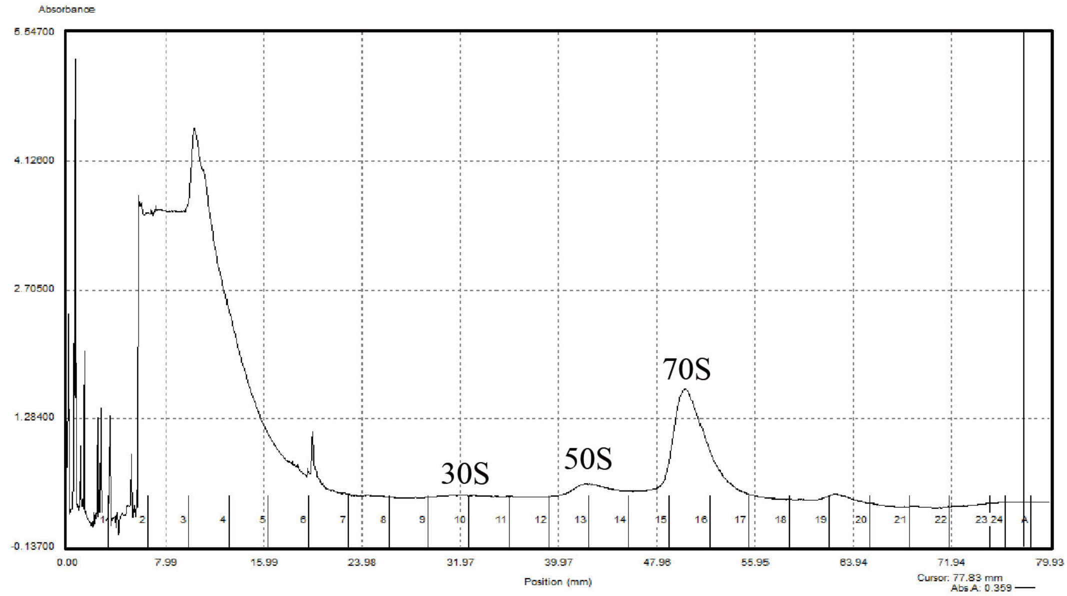

Supplementary Figure S1. Sucrose gradient separation of the ribosomal material from *M. smegmatis* BRkoA.

Supplementary Table S1:

Strains used in this study.

Species	Strains	Characteristics	Sources
Escherichia coli	DH10B	Host strain for cloning	Invitrogen
Mycobacterium smegmatis	MC^2 155	Wild-type	Lab stock
	koA	MC^2 155 Δ*MSMEG_3755-3757*, *Ms rrnA* gene was knocked out, *Hyg*^{R}	Lab stock
	koB	MC^2 155 Δ*MSMEG_4929-4931*, *Ms rrnB* gene was knocked out, *Hyg*^{R}	This work
	BR	koB derivate, with complete *Mt rrn* gene (*Rvnr01-03*) from *Mycobacterium tuberculosis* H37Rv downstream of gene *MSMEG_4932*, *Kan*^{R}, ϕC31_*attB* and *attP*	This work
	BRkoA-Apra^R	BR derivate, *ΔMSMEG_3755-3757*，*Ms rrnA* gene was knocked out, Cint, *Apra*^{R}, TG1_*attB* and *attP*	This work
	BRkoA-Hyg^R	BRkoA-Apra^R derivate, harboring pCQ1	This work
	BRkoA	BRkoA-Hyg^R derivate, without pCQ1	This work

Supplementary Table S2:

plasmids used in this study.

Plasmids	Characteristics	Sources
pXL1	*ori p15A*, *Bint*, *Kan*^{R}, ϕC31_*attB* and *attP*, *SacB*	Lab stock
pXL2	*ori p15A*, *Cint*, *Apra*^{R}, TG1_*attB* and *attP*, *SacB*	Lab stock
pXL3	*ori p15A*, *TGint*, *Hyg*^{R}, BT1_*attB* and *attP*, *SacB*	Lab stock
pXL12	*Cint*, *Apra*^{R}, *ori p15A*, *oriM*^{ts}	Lab stock
pQX03	*Bint*, *Hyg*^{R}, *Amp*^{R}, BT1_*attP* and ϕC31_*attB*, H37Rv-rRNA integration	Lab stock
pCQ1	*ori p15A*, *Hyg*^{R}, *TGint*, *oriM*^{ts}	This work
pCQ4	*Hyg*^{R}, *SacB*, *Amp*^{R}, upstream and downstream homologous fragment of MC^2 155 *MSMEG_4929-4931* (*Ms rrnB* gene)	This work
pCQ6	*Kan*^{R}, ϕC31_*attB* and *attP*, *SacB*, upstream and downstream homologous fragment of MC^2 155 *MSMEG_4929-4931* (*Ms rrn*B gene), complete *Mt rrn* gene (*Rvnr01-03*) from *Mycobacterium tuberculosis* H37Rv	This work
pCQ7	*Cint*, *Apra*^{R}, *Amp*^{R}, TG1_*attB* and *attP*, *SacB*, upstream and downstream homologous fragment of MC^2 155 *MSMEG_3755-3757* (*Ms rrnA* gene)	This work

Supplementary Table S3:

Primers used in this study for construct the corresponding plasmids or Sanger sequencing.

Products (Sizes)	Template	Primer	Sequence (5′ → 3′)
pCQ1-*ori*Mts (3586 bp)	pXL12	*ori*Mts-F	CAAAGGAATAGGGTGCTGGGAAACAAACCACCGCTGGTAG
		*ori*Mts-R	TCGGAATCGCAGACCGATAC
pCQ1-TGint-*ori* p15A-Hyg (4186 bp)	pXL3	Hyg-TG1-F	GTATCGGTCTGCGATTCCGACTAGCTTGCAGTGGGCTTAC
		Hyg-TG1-R	CCCAGCACCCTATTCCTTTG
pCQ4-ΔB-left flank (2028 bp)	MC² 155	ΔB-Up-F	GCATTGGTAACTCGAGCTGTGGGTTCTCCGGGTTCAAGAC
		ΔB-Up-R	ACCGCCGAACACAAATTGAC
pCQ4-ΔB-right flank (2013 bp)	MC² 155	ΔB-Down-F	GTCCGAGGGCAAAGGAATAGCAACCCATCCAAAGACAGGT
		ΔB-Down-R	GCGGGTACTTCGGATCTTCG
pCQ4-ΔB-*SacB* (2115 bp)	pXL3	ΔB-*SacB*-F	CGAAGATCCGAAGTACCCGCCCGCACCCATCACATATACC
		ΔB-*SacB*-R	CTGATATGCCGCCCGGTAGT
pCQ4-ΔB-Amp (1050 bp)	pQX03	ΔB-Amp-F	ACTACCGGGCGGCATATCAGGTACCACGGCTAATGGTTTC
		ΔB-Amp-R	ACAGCTCGAGTTACCAATGC
pCQ4-ΔB-*ori* p15A&Hyg (2116 bp)	pCQ1	ΔB-*ori* p15A&Hyg-F	GTCAATTTGTGTTCGGCGGTACTCGAGCTGTCAGACCAAG
		ΔB-*ori* p15A&Hyg-R	CTATTCCTTTGCCCTCGGAC
pCQ6-BR-left flank (2028 bp)	MC² 155	B-Up-F	CGTACAATTGGATGCGGTTGACCGCCGAACACAAATTGAC
		B-Up-R	GGGTTCTCCGGGTTCAAGAC
pCQ6-BR-right flank (2013 bp)	MC² 155	B-Down-F	GGTATATGTGATGGGTGCGGGCGGGTACTTCGGATCTTCG
		B-Down -R	CAACCCATCCAAAGACAGGT
pCQ6-BR-*SacB* (2126 bp)	pXL3	B-*SacB*-F	GTCTTGAACCCGGAGAACCCTGCCTTGGATCCTGATATGC
		B-*SacB*-R	CCGCACCCATCACATATACC
pCQ6-BR-*Mt rrn* (5308 bp)	pQX03	B-*Mt rrn*-F	ACCTGTCTTTGGATGGGTTGTTTTGTTTGGAGAGTTTGAT
		B-*Mt rrn*-R	GCGCCCCCGCCCGCGAGGGT
pCQ6-BR-*ori* p15A&Kan (2059 bp)	pXL1	B-*ori* p15A&Kan-F	ACCCTCGCGGGCGGGGGCGCCCTGGTGTCCCTGTTGATAC
		B-*ori* p15A&Kan-R	CAACCGCATCCAATTGTACG
pCQ7-ΔA-left flank (2137 bp)	MC² 155	ΔA-Up-F	GCATTGGTAACTCGAGCTGTTAGCGTCATCAACGCTAGTC
		ΔA-Up-R	GCCGATGATACTACCCTTCC
pCQ7-ΔA-right flank (2087 bp)	MC² 155	ΔA-Down-F	CCATATGAGTTCGGTTGCGTCAACCCATCCAAAGACAGGT
		ΔA-Down-R	GTATGCGCCAATGTCGTGTG
pCQ7-ΔA-*SacB*&Amp (3145 bp)	pCQ4	ΔA-*SacB*&Amp-F	CACACGACATTGGCGCATACCCGCACCCATCACATATACC
		SacB&Amp-R	ACAGCTCGAGTTACCAATGC
pCQ7-ΔA-ϕC31-*ori* p15A&Apar (3946 bp)	pXL2	ΔA-ϕC31-*ori* p15A&Apar-F	GGAAGGGTAGTATCATCGGCATCCCGCCCAACTGGGTAAG
		ΔA-ϕC31-*ori* p15A&Apar-R	ACGCAACCGAACTCATATGG
Primers used for sequencing pCQ1		pCQ1-YZ-1	GTCCGAGGGCAAAGGAATAG
		pCQ1-YZ-2	CCACGTCGCCGACGAATTTG
		pCQ1-YZ-3	ACGGGATCGTGGTCTACGAG
		pCQ1-YZ-4	GTGATGTACGTGGCGAACTC

	pCQ1-YZ-5	CCCGTCAAGTCAGCGTAATG
	pCQ1-YZ-6	GACATTATTCCGGTCGTCAC
	pCQ1-YZ-7	ATCGCTCCTGTCGGCAATGG
Primers used for sequencing pCQ4	pCQ4-YZ-1	CCTCCCGTATCGTAGTTATC
	pCQ4-YZ-2	GCTTCCGCAACCTCACCAAC
	pCQ4-YZ-3	GTCGGCTCATCGAACATTCC
	pCQ4-YZ-4	CTGTCGGGCGTACACAAATC
	pCQ4-YZ-5	AGCCGCGATCGATGTGGAAC
	pCQ4-YZ-6	GCGGCCATCAGGATGTTCTC
	pCQ4-YZ-7	ATTTCAGCCGCGTGCCCAAG
	pCQ4-YZ-8	AAGAAGCAGACCGCTAACAC
	pCQ4-YZ-9	AGCTCAGGCGACAACCATAC
Primers used for sequencing pCQ6	pCQ6-YZ-1	GTTGGCTACCCGTGATATTG
	pCQ6-YZ-2	ATAGGTCTGGTCCTCCATAC
	pCQ6-YZ-3	TGTGTCTGACGGGAAAGATG
	pCQ6-YZ-4	CTGCCTTGGATCCTGATATG
	pCQ6-YZ-5	AGCGCCGTTTGCTAACTCAG
	pCQ6-YZ-6	TTTGTAATGGCCAGCTGTCC
	pCQ6-YZ-7	GTGAACGGCAGGTATATGTG
	pCQ6-YZ-8	AAGTACGACGCCGACTTTGC
	pCQ6-YZ-9	GACGGGACGTCGATGATCAC
	pCQ6-YZ-10	AAGTCGAACGGAAAGGTCTC
	pCQ6-YZ-11	GTCTCTGGGCAGTAACTGAC
	pCQ6-YZ-12	GATTGGGACGAAGTCGTAAC
	pCQ6-YZ-13	CCCGGTAAGCCTAAATACTC
	pCQ6-YZ-14	GTGATTGTGCGCCGATAATG
	pCQ6-YZ-15	AGTGAGGAGCGACTGTTTAC
	pCQ6-YZ-16	GGGCTGTTCGCCCATTAAAG
Primers used for sequencing pCQ7	pCQ7-YZ-1	TAGCCGTGGTACCTGATATG
	pCQ7-YZ-2	AGCGCCGTTTGCTAACTCAG
	pCQ7-YZ-3	TTTGTAATGGCCAGCTGTCC
	pCQ7-YZ-4	AGACTACGGGCCTAAAGAAC
	pCQ7-YZ-5	GTCTATTCCGGGTTCGATCC
	pCQ7-YZ-6	AGATGACCAGCCCGTATGCC
	pCQ7-YZ-7	CTCGCTGCCACTAGAGAATG
	pCQ7-YZ-8	TTGACTGCCCGGCTTGAAGG
	pCQ7-YZ-9	CTTCGGCGTGCCTGATCTTG
	pCQ7-YZ-10	GGTCTGACGCTCAGTGGAAC
	pCQ7-YZ-11	TGCCTTTGCTCGGTTGATCC
	pCQ7-YZ-12	AACTCGTGACCCTGGACAAG
	pCQ7-YZ-13	GCAGGTCGCCGAACATCTAC

Supplementary Table S4:

Primers used in this study for validate the corresponding strains or Sanger sequencing.

Products (Sizes)	Primer	Sequence (5′ → 3′)
koB-Up (2785 bp)	ΔB-Up-YZ-F	CACGGTGTGATGCGTTATCG
	ΔB-Up-YZ-R	GCTTGGTTGACGGCAATTTC
koB-Down (3144 bp)	ΔB-Down-YZ-F	CGCTCCTCCAAGCCAGTTAC
	ΔB-Down-YZ-R	TGCACCGTGACACTGAAATG
koB-Target gene (2499 bp)	ΔB-Target-YZ-F	GTCCAAGAGTGCGGCAGTGA
	ΔB-Target-YZ-R	GGAAGTCTCACGGGTTATGG
BR-Up (2819 bp)	BR-Up-YZ-F	CACGGTGTGATGCGTTATCG
	BR-Up-YZ-R	CTCATCCCACACCGCTAAAG
BR-Down (3065 bp)	BR-Down-YZ-F	GTTGGCTACCCGTGATATTG
	BR-Down-YZ-R	TGCACCGTGACACTGAAATG
BR-Target gene (1186 bp)	BR-Target-YZ-F	CCTGTTCGGTTCGTAAACTG
	BR-Target-YZ-R	CGGCGAGTACTTCTACACAG
BRkoA-Apra^R-Up (2862 bp)	BRkoA-Up-YZ-F	CTCCTCCGGTTGTACTTCTG
	BRkoA-Up-YZ-R	CGTACGTGTCCATGCGAAAC
BRkoA-Apra^R-Down (2523 bp)	BRkoA-Down-YZ-F	GAGGCGGGATGCGAAGAATG
	BRkoA-Down-YZ-R	TCCGGCACCGATCAACTCAG
BRkoA-Apra^R-Target gene (3826 bp)	BRkoA-Target-YZ-F	GCACAGCAAACGTTGAGATG
	BRkoA-Target-YZ-R	CACTGTCTCCGAAAGGATAC
BRkoA-Apra^R-Kan resistance gene (2409/456 bp)	BRkoA-Kan-YZ-F	TTCCGAACCCGGAAGCTAAG
	BRkoA-Kan-YZ-R	GGAAGTCTCACGGGTTATGG
BRkoA-Hyg^R-Apra resistance gene (4605/745 bp)	BRkoA-Apra-YZ-F	CGACGAGTGGGTACCACAAG
	BRkoA-Apra-YZ-R	CACTGTCTCCGAAAGGATAC
BRkoA-B (5737 bp)	BRkoA-B-YZ-F	GCGACGAACCTCGTATTATC
	BRkoA-B-YZ-R	GGAAGTCTCACGGGTTATGG
BRkoA-A (745 bp)	BRkoA-A-YZ-F	CGACGAGTGGGTACCACAAG
	BRkoA-A-YZ-R	CACTGTCTCCGAAAGGATAC
sigA (78 bp)	qPCR-*sigA*-F	CCACCGGGAATTCGTAAGAC
	qPCR-*sigA*-R	CTTCACCGGCTCTTCGGTTG
rrn (181/184 bp)	qPCR-*rrn*-F	GGTGCTGGAAGGTTAAGAGG
	qPCR-*rrn*-R	TTTCGCCGAGTCTATGGTTG
